# The associations of body mass index, bioimpedance spectroscopy-based calf intracellular resistance, single-frequency bioimpedance analysis and physical performance of older people

**DOI:** 10.1007/s40520-019-01301-8

**Published:** 2019-08-29

**Authors:** Mikko P. Björkman, Satu K. Jyväkorpi, Timo E. Strandberg, Kaisu H. Pitkala, Reijo S. Tilvis

**Affiliations:** 1 Geriatric Unit, Department of Internal Medicine, Institute of Clinical Medicine, University of Helsinki, POB 20, 00014 Helsinki, Finland; 2 Department of General Practice and Primary Health Care, University of Helsinki, Helsinki, Finland; 3 Center for Life Course Health Research, University of Oulu, Oulu, Finland

**Keywords:** Bioelectrical impedance, Intracellular resistance, Muscle strength, Physical performance, Sarcopenia

## Abstract

**Background:**

Bioimpedance skeletal muscle indices (SMI) are used as a surrogate for skeletal muscle mass, but their associations with physical functioning and obesity need further evaluation.

**Aims:**

To compare the associations of body mass index (BMI), bioimpedance spectroscopy-based calf intracellular resistance (Cri-SMI), and single-frequency bioimpedance analysis (SF-SMI) indices with physical performance and the functioning of community-dwelling older people at risk of or already suffering from sarcopenia.

**Methods:**

Pre-intervention measurements of the screened subjects and the participants of the Porvoo sarcopenia trial (*N *= 428) were taken. Cri-SMI, whole-body SF-SMI, and BMI were related to hand-grip strength, walking speed, short physical performance battery (SPPB), and the physical component of the RAND-36.

**Results:**

Among the older people (aged 75–96), Cri-SMI correlated inversely with age (men *r *= - 0.113, *p *< 0.001; women *r *= - 0.287, *p *< 0.001), but positively with SPPB (*r *= 0.241, *p *< 0.001) and the physical component of the RAND-36 (*r *= 0.114, *p *= 0.024), whereas BMI was inversely associated with SPPB (*r *= - 0.133, *p *< 0.001) and RAND-36 (*r *= - 0.286, *p *< 0.001). After controlling for age, gender, and comorbidity, one unit of Cri-SMI (cm^2^/Ω) was associated with a 3.3-fold probability of good physical performance (SPPB ≥ 9 points, OR = 3.28, *p *< 0.001) and one unit of BMI (kg/m^2^) decreased the respective probability 4% (OR= 0.96, *p *= 0.065). Physical inactivity partly explained the negative association of BMI. When Cri-SMI and BMI were controlled for, a 1% difference in Cri-SMI was associated with a 0.7% (*p *< 0.001) higher probability of good performance, the respective figure being - 2.2% (*p *= 0.004) for BMI. The associations of SF-SMI with physical functioning indices were insignificant.

**Conclusions:**

Independent of each other, Cri-SMI was positively and BMI was inversely associated with the physical performance and functioning of community-dwelling older people who were at risk of or already suffering from sarcopenia. We found no association between SF-SMI and physical functioning.

## Introduction

Age-related muscle loss, i.e., sarcopenia is a significant contributor to declining physical functioning and reduced quality of life among older people [1, 2]. According to a recent consensus definition, sarcopenia is determined by muscle strength, muscle quality and quantity, and physical performance [3]. This creates a need to measure the muscle mass of heterogeneous older populations in a wide range of clinical settings. Different body imaging techniques, such as computed tomography, magnetic resonance imaging or dual-energy X-ray absorptiometry (DEXA), have been recommended for the measurement of muscle mass [3]. However, these imaging techniques require a visit to a laboratory or a hospital, which may present difficulties in availability and be problematic for some older sarcopenic people with disabilities. Bioimpedance analysis (BIA) is a portable alternative for assessing body composition [4, 5] and is thus suitable for primary health care settings, including home visits. However, the accuracy of algorithm-based single-frequency BIA (SF-BIA) in the assessment of the muscle mass of some older population, in particular, has been questioned [6–8]. SF-BIA estimates of muscle mass are based on algorithms that are often derived from relatively healthy subjects, and so may be a source of inaccuracy among older multimorbid and disabled people. Furthermore, it has been suggested that the excess extracellular water in muscles may mask actual muscle atrophy during aging [9, 10]. This extracellular water compartment may result in the overestimation of actual muscle mass when using SF-BIA and imaging techniques.

Bioimpedance spectroscopy (BIS) offers an alternative method for investigating muscle biology [6]. It uses hundreds of frequencies within a wide range, allowing the calculation of intracellular resistance (Ri), a measure that does not require participant characteristic data or population-based algorithms. Ri is closely related to the intracellular water (ICW) compartment and may be considered a surrogate for skeletal muscle cell mass, as fat and bone cells have a low content of intracellular water [6, 10]. Recent studies by Yamada et al. have underscored the value of BIS as a measure of muscle function and for providing information on skeletal muscle biology [11].

Changes in segmental calf intracellular skeletal muscle index (Cri-SMI) have shown to be associated with mobility decline among typical nursing-home residents [12]. We investigated the associations of BMI and two bioelectrical impedance skeletal muscle indices with the physical performance of community-dwelling older people who were at risk of or already suffering from sarcopenia. Our second aim was to evaluate the interplay of muscle mass and BMI as associates of the physical performance and functioning of old people.

## Methods

This cross-sectional study is based on the baseline screening data of the Porvoo sarcopenia and Nutrition trial (ACTRN12612001253897). The trial procedures have been published elsewhere [13]. We approached the population aged 75 +  living in Porvoo, Finland (*N *= 3275) by a postal questionnaire (response rate 60.5%) and the research group further examined the individuals at risk of sarcopenia (limitations in daily living activities, sedentary lifestyle, falls, exhaustion, old age, low body mass index (BMI)). The key exclusion criteria were not living at home, not being able to walk indoors independently (canes and walkers were allowed), and not being able to cooperate with bioimpedance and hand-grip strength measurements. We also excluded patients with cardiac pacemakers and severe skin lesions in bioimpedance electrode placement sites. The study protocol was approved by the ethics committee for internal medicine of the hospital district of Helsinki and Uusimaa. We obtained informed consent from each patient or their next of kin. Participants signed an informed consent form before beginning any trial procedures. In the case of participants’ cognitive decline (Mini-mental state sxamination (MMSE) < 19) [14] or poor judgment ability, we invited a proxy to give consent in addition to the participant’s consent.

We collected the demographic data and medical history via the postal questionnaire. The questionnaires included the RAND-36 physical functioning scale [15, 16]. Patients who reported being able to walk less than 1 km per day on average or exercising regularly for less than 1 h per week were classified as physical inactive. Morbidity was assessed using the Charlson comorbidity index [17] and muscle endurance by the 2-min step test [18]. We also calculated BMI.

The participants were examined at a day clinic or during a home visit. Physical performance was assessed by the short physical performance battery (SPPB) [19], which includes a three-part balance test, habitual gait speed, and a chair stand test. Each category of SPPB is scored from 0 to 4, so the total score ranges from 0 to 12, with 0 indicating poorest and 12 indicating best performance. Muscle strength was assessed by a hand-grip dynamometer (JAMAR dynamometer, Saehan Corp., Masan, Korea). We recorded the mean maximum strength of both hands, as well as 4-m course habitual gait speed as a part of SPPB with a cut-off point  < 0.8 m/s. We evaluated cognitive functioning using the mini-mental state examination (MMSE) [14], with a score ranging from 0 (poorest) to 30 (best).

We preformed bioimpedance spectroscopy by a single-channel, tetra-polar device (SFB7, ImpediMed Ltd., Eight Miles Plains, Queensland, Australia) that scans 256 frequencies between 4 and 1000 kHz. The values were recorded without further software processing. The Cri-SMI was calculated from the BIS data of the calf measurements as follows: Cri-SMI = electrode distance^2^/Ri_calf_ (cm^2^/Ω), using the means of both calves. Finally, the whole-body single-frequency skeletal muscle index (SF-SMI) was calculated from the whole-body skeletal muscle mass (SMM), and assessed according to Janssen et al. [5]. We then transformed this into a skeletal muscle index as follows: SF-SMI = SMM/height^2^.

We used SPSS software (IBM Corp. Released 2012. IBM SPSS Statistics for Windows, Version 21.0. Armonk, NY: IBM Corp.) for the statistical analyses. Continuous variables with normal distribution were expressed by means of standard deviations (SD). For the variables with normal distribution, statistical comparisons between the groups were made using Student’s *t* test and for those with skewed distribution the Mann–Whitney *U* test. We used the Chi-square test to examine the relationship between the two categorical variables and used Fisher’s exact test when appropriate. Pearson’s correlation coefficient was used to describe the bivariate correlations between the normally distributed variables and Spearman’s rho for the variables with a skewed distribution. Logistic regression models were created to calculate the unadjusted and adjusted odds ratios and 95% confidence intervals. *p* values below 0.050 were considered statistically significant.

## Results

The participants (*N *= 428) were old (83.4 years); women outnumbered men (285 vs 143); and most (56%) of them lived alone. They used a mean of 5.4 prescribed regular medications. The mean SPPB score was 8.2, and 54.8% of the participants scored at least 9 points. The men had higher Charlson comorbidity indices (Table 1), larger muscle mass indices, and stronger hand-grip strength than the women. The men also tended to have better physical functioning (RAND-36) (Table 1).

**Table 1:** Selected baseline characteristics (SD) of participants by gender

Variable	Women (*N *= 285)	Men (*N *= 143)	*p* value
Age, years	83.4 (4.7)	83.5 (4.3)	0.87
Charlson Comorbidity Index (CCI)	2.0 (1.7)	2.7 (2.3)	< 0.001
MMSE	25.9 (3.2)	25.2 (3.9)	0.059
BMI, kg/m^2^	27.4 (4.7)	26.8 (4.0)	0.25
SPPB (0–12)	8.3 (3.0)	8.2 (3.0)	0.80
RAND-SF (0–100)	48.4 (26.7)	54.2 (30.1)	0.051
2-min walking, m	76.3 (24.3)	79.0 (26.6)	0.30
Grip strength, kg	17.6 (4.5)	28.9 (7.0)	< 0.001
SF-SMI, kg/m^2^	6.9 (1.0)	9.7 (1.2)	< 0.001
Cri-SMI, cm^2^/Ω	1.4 (0.4)	1.7 (0.5)	< 0.001

*RAND*-*SF* physical functioning scale of RAND-36, *MMSE* mini-mental state examination, *BMI* body mass index, *SF*-*SMI* single-frequency skeletal muscle index, *Cri*-*SMI* segmental calf intracellular resistance skeletal muscle index

**Fig. 1:**
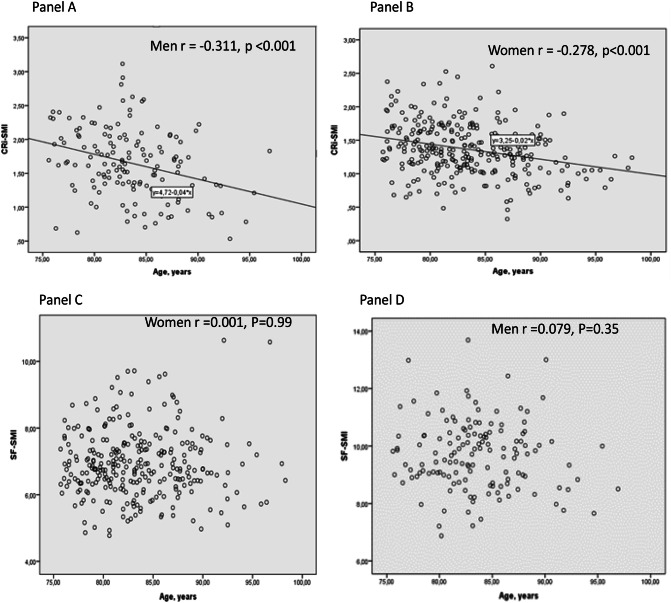
Correlations of age with Cri-SMI and SF-SMI among men and women

We first investigated the relationship of muscle mass measures with age, BMI, and physical functioning. Cri-SMI correlated inversely with age among both the men and the women, whereas SF-SMI did not (Fig. 1). BMI correlated positively with Cri-SMI and SF-SMI, but inversely with physical performance and functioning indices (Table 2). Controlled-for age and gender Cri-SMIs were associated with good physical performance and functioning including walking speed, whereas the respective figures were insignificant for SF-SMI. We found significant, although rather weak, correlations between grip strength and BMI, muscle mass indices and physical functioning scores.

**Table 2:** Age- and gender-adjusted intercorrelations *(p* values*)* of body mass index, bioimpedance muscle indices, and physical functioning (*N *= 428)

	BMI	SF-SMI	Cri-SMI	RAND-36	SPPB	Walking speed	Grip strength
BMI	1	< *0.001*	< *0.001*	< *0.001*	*0.008*	< *0.001*	*0.008*
SF-SMI	0.573	1	< *0.001*	*0.029*	*0.240*	*0.105*	< *0.001*
CRi-SMI	0.346	0.542	1	*0.024*	< *0.001*	*0.005*	< *0.001*
RAND-36	− 0. 286	− 0.109	0.114	1	< *0.001*	< *0.001*	< *0.001*
SPPB	− 0.133	− 0.059	0.241	0.580	1	< *0.001*	< *0.001*
Walking speed	− 0.181	− 0.082	0.141	0.505	0.505	1	*0.004*
Grip strength	0. 133	0.197	0.299	0.191	0.249	0.144	1

*P* values are in italics

*RAND*-*SF* physical functioning scale of RAND-36, *MMSE* mini-mental state examination, *BMI* body mass index, *SF*-*SMI* single-frequency skeletal muscle index, *Cri*-*SMI* segmental calf intracellular resistance skeletal muscle index

When the study population was grouped by CRi-SMI cut-off points, the individuals with low values were characterized by older age, lower BMI, weaker hand-grip strength, physical inactivity, and lower physical performance and functioning scores (Table 3). The differences were similar among both the men and the women. Closer analysis showed that low Cri-SMI was negatively associated (*p *= 0.002) with SPPB quartiles among both the men and the women. Again, we found no significant association for SF-SMI (*p *= 0.778).

**Table 3:** Characteristics of community-dwelling older people at risk of sarcopenia by calf intracellular resistance skeletal muscle index (Cri-SMI)

	Women (Cri-SMI cut-off point 1.50)			Men (Cri-SMI cut-off point 2.06)			Both (Gender-specific cut-off points)		
	Normal Cri-SMI	Low Cri-SMI	*p* value	Normal Cri-SMI	Low Cri-SMI	*p* value	Normal Cri-SMI	Low Cri-SMI	*p* value
Number	96	187		31	111		127	298	
							75.6	62.8	0.010
Age. years (± SD)	82.0 ± 4.0	84.1 ± 5.0	< 0.001	81.7 ± 3.7	84.0 ± 4.4	0.009	81.9 ± 3.9	84.1 ± 4.8	< 0.001
Charlson comorbidity index (range)	1.0 (1.0–2.0)	2.0 (1.0–3.0)	0.288	2.0 (1.0–4.0)	2.0 (1.0 to − 4.0)	0.153	2 (1–3)	2 (1–3)	0.700
Sedentary lifestyle (%)	32.3	50.3	0.004	26.7	33.0	0.507	31	43.8	0.014
Limitations walking indoors (%)	12.5	21.0	0.080	19.4	22.9	0.672	14.2	21.7	0.073
At least one fall in past 12 months (%)	32.6	36.3	0.543	46.7	45.4	0.900	36.0	39.7	0.476
Physical function scale of RAND-36 mean points (± SD)	54.3 ± 24.3	45.7 ± 27.3	0.011	55.3 ± 27.9	54.3 ± 30.7	0.865	55 (35–75)	50 (25–75)	0.061
Short physical performance battery (range)	10 (8–11)	8 (6–11)	0.001	10 (8–12)	8 (6–10)	0.017	10 (8–11)	8 (6–10)	< 0.001
Habitual gait speed m/s (± SD)	0.95 ± 0.28	0.84 ± 0.33	0.004	0.93 ± 0.30	0.89 ± 0.32	0.557	0.95 ± 0.28	0.86 ± 0.33	0.008
Walking aid in gait speed test (%)	1.0	4.3	0.281	0.0	3.6	0.284	0.79	4.0	0.120
Grip strength kg (± SD)	19.2 ± 4.1	16.6 ± 4.3	< 0.001	30.5 ± 7.1	27.1 ± 6.9	0.017	20.5 (17.5–25)	19.5 (16–24.5)	0.034
Mini-mental state examination (range)	27 (25–28)	26 (24–28)	0.379	27 (24–28)	26 (23–28)	0.363	27 (25–28)	26 (24–28)	0.166
Body mass index. kg/m^2^ (± SD)	29.3 ± 4.9	26.4 ± 4.7	< 0.001	28.5 ± 3.8	26.3 ± 3.9	0.006	29.1 ± 4.6	26.4 ± 4.4	< 0.001
SF-SMI^a^. kg/m^2^ (± SD)	7.6 ± 0.9	6.6 ± 0.9	< 0.001	10.5 ± 1.3	9.5 ± 1.1	< 0.001	7.8 (7.1–9.3)	7.3 (6.4–9.0)	< 0.001

Means ± standard deviations or medians with first and fourth quartile cut-off points or percentages

*SD* standard deviation, *SF*-*SMI*^*a*^ single frequency bioimpedance analysis

^a^Whole-body single-frequency skeletal muscle index

**Fig. 2:**
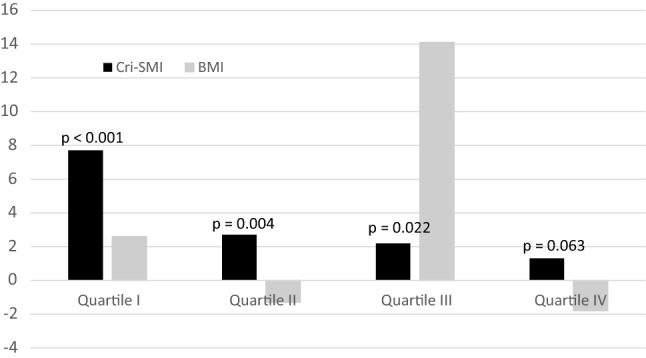
Associations of Cri-SMI and BMI by BMI quartiles with good physical performance (SPPB > 9). Quartile cut points: 23.95, 26.58, and 29.73 kg/m^2^

Finally, the opposite relationships of BMI and Cri-SMI to physical functioning scores prompted us to test the nature of these associations in logistic regression analyses (Table 4). After controlling for age and gender, one unit of Cri-SMI was associated with a 3.3-fold probability that SPPB was at least 9 points, whereas one unit of BMI decreased the respective probability by 4% (OR= 0.96). Further controlling for comorbidity and physical inactivity did not substantially weaken these associations. However, physical inactivity partly explained the negative association with BMI. The odds ratios of both Cri-SMI (OR= 4.4) and BMI (OR= 0.92) became even stronger when controlled for each other. Due to the large differences in the scales of Cri-SMI and BMI, we used their percentage values (mean value = 100%) for comparisons. When Cri-SMI and BMI were controlled for age, gender, comorbidity, physical activity, and for each other, a 1% difference in Cri-SMI was associated with a 0.7% (*p *< 0.001) increase in the probability of good performance, the respective figure being - 2.2% (*p *= 0.004) for BMI. The respective associations of SF-SMI with physical functioning indices were insignificant. Finally, when patients were classified according to BMI quartiles, the associations of Cri-SMI with good performance were strongest in the persons with low BMI while those of BMI were inconsistent (Fig. 2).

**Table 4:** Associations of Cri-SMI and BMI with good physical performance (SPPB ≥ 9)

Cri-SMI				BMI				
Unit	OR	95% CIs	*p* value	Unit		OR	95% CIs	*p* value
Unadjusted								
cm^2^/Ω	3.64	2.25–5.89	< 0.001	kg/m^2^	0.95		0.91–0.99	0.012
%	1.006	1.004–1.008	< 0.001	%	0.987		0.98–1.00	0.026
Adjusted for age and gender								
cm^2^/Ω	3.38	1.99–5.73	< 0.001	kg/m^2^	0.96		0.91–0.99	0.039
%	1.006	1.003–1.008	< 0.001	%	0.987		0.77–1.00	0.039
Adjusted for age, gender, and comorbidity								
cm^2^/Ω	3.28	1.92–5.60	< 0.001	kg/m^2^	0.96		0.91–0.99	0.065
%	1.005	1.003–1.008	< 0.001	%	0.989		0.98–1.00	0.065
Adjusted for age, gender, comorbidity, and physical inactivity								
cm^2^/Ω	3.17	1.80–5.51	< 0.001	kg/m^2^	0.97		0.93–1.02	0.198
%	1.005	1.003–1.008	< 0.001	%	0.992		0.98–1.00	0.198
Adjusted for age, gender, comorbidity, physical inactivity, and BMI				Adjusted for age, gender, comorbidity, physical inactivity, and Cri-SMI				
cm^2^/Ω	4.42	2.39–8.22	< 0.001	kg/m^2^	0.92		0.87–0.97	0.004
%	1.007	1.004–1.010	< 0.001	%	0.98		0.96–0.99	0.004

*BMI* body mass index, *Cri*-*SMI* segmental calf intracellular resistance skeletal muscle index

## Discussion

This cross-sectional study shows that Cri-SMI is significantly associated with good physical functioning, whereas BMI is related to poor physical functioning among community-dwelling older people. We found no association between SF-SMI and physical functioning. Our results also demonstrate that the relationships between BMI, bioimpedance skeletal muscle indices, and physical functioning scores are rather linear and the associations are “dose-dependent.” Accordingly, the results are not affected by the cut-off points selected for test variables. Our data also shed new light on the interplay between age, muscle mass, physical activity, and obesity among older people. Cri-SMI and BMI were both independent associates of physical performance.

These observations support our previous longitudinal nursing-home study, which showed an association between Cri-SMI change, mobility decline, and need of help [12]. The results are also in good accordance with the data of the recent Kyoto–Kameoka Study, in which thigh BIS was a strong predictor of knee extension strength and gait speed was independent of age, sex, body mass index, and muscle mass among community-dwelling people aged between 65 and 90 [20].

The results provide a plausible explanation for the difference between the associations of Cri-SMI and SF-SMI. The relatively strong correlation between BMI and SF-SMI (*r *= 0.573, *p *< 0.001) suggests that the possible positive relationship between SF-SMI and physical functioning is effectively masked by BMI having a negative influence. It is also important to note that Cri-SMI is a direct measurement, whereas SF-SMI is calculated using both height and weight. This may hamper the value of SF-SMI in the evaluation of the interplay between muscle mass and obesity.

Muscle atrophy during aging decreases the intracellular compartment of the muscle, but the extracellular fluid is maintained, resulting in an increase in the proportion of non-functional muscle volume [20]. This may mask age-related muscle loss. The use of intracellular resistance may diminish the confounding effect of extracellular fluid in muscle tissue. This could explain the lack of association between SF-SMI and physical performance. It should be noted that SPPB mainly measures the physical performance of the lower limbs (gait speed, chair stand, and balance). SF-SMI utilized whole-body bioimpedance measurements, whereas Cri-SMI was calculated from calf data, which may strengthen its association with lower limb performance-based SPPB. We did not find any significant new gender differences that should be taken into account when interpreting bioimpedance data (data not shown).

The main weakness of this study is its lack of confirmatory skeletal muscle measurements (dual X-ray absorptiometry, etc.). Its strengths are the good representatives of the population sample. Home visits were offered to ensure the participation of true geriatric patients. We used well-validated tools to assess physical performance. Clinically important confounders were recorded, and their influences were tested in multiple ways. Finally, to the best of our knowledge, this is the second and largest study to investigate the role of Cri-SMI in the physical performance of community-dwelling older people.

## Conclusions

This study shows that Cri-SMI is associated positively and BMI inversely with the physical functioning of community-dwelling older people at risk of sarcopenia. Whole-body SF-SMI correlated with BMI, but not with physical functioning scores.
